# Low-Density Lipoprotein Receptor-Related Protein 1 (LRP1) Mediates Neuronal Aβ42 Uptake and Lysosomal Trafficking

**DOI:** 10.1371/journal.pone.0011884

**Published:** 2010-07-29

**Authors:** Rodrigo A. Fuentealba, Qiang Liu, Juan Zhang, Takahisa Kanekiyo, Xiaoyan Hu, Jin-Moo Lee, Mary Jo LaDu, Guojun Bu

**Affiliations:** 1 Department of Pediatrics, Washington University School of Medicine, St. Louis, Missouri, United States of America; 2 Department of Neurology, Washington University School of Medicine, St. Louis, Missouri, United States of America; 3 Department of Anatomy and Cell Biology, University of Illinois at Chicago, Chicago, Illinois, United States of America; 4 Department of Cell Biology and Physiology, Washington University School of Medicine, St. Louis, Missouri, United States of America; New York State Institute for Basic Research, United States of America

## Abstract

**Background:**

Alzheimer's disease (AD) is characterized by the presence of early intraneuronal deposits of amyloid-β 42 (Aβ42) that precede extracellular amyloid deposition in vulnerable brain regions. It has been hypothesized that endosomal/lysosomal dysfunction might be associated with the pathological accumulation of intracellular Aβ42 in the brain. Our previous findings suggest that the LDL receptor-related protein 1 (LRP1), a major receptor for apolipoprotein E, facilitates intraneuronal Aβ42 accumulation in mouse brain. However, direct evidence of neuronal endocytosis of Aβ42 through LRP1 is lacking.

**Methodology/Principal Findings:**

Here we show that LRP1 endocytic function is required for neuronal Aβ42 uptake. Overexpression of a functional LRP1 minireceptor, mLRP4, increases Aβ42 uptake and accumulation in neuronal lysosomes. Conversely, knockdown of LRP1 expression significantly decreases neuronal Aβ42 uptake. Disruptions of LRP1 endocytic function by either clathrin knockdown or by removal of its cytoplasmic tail decreased both uptake and accumulation of Aβ42 in neurons. Finally, we show that LRP1-mediated neuronal accumulation of Aβ42 is associated with increased cellular toxicity.

**Conclusions/Significance:**

These results demonstrate that LRP1 endocytic function plays an important role in the uptake and accumulation of Aβ42 in neuronal lysosomes. These findings emphasize the central function of LRP1 in neuronal Aβ metabolism.

## Introduction

Alzheimer's disease (AD) is a neurodegenerative disorder of the central nervous system characterized by a progressive decline in cognitive functions and neuronal loss. AD was originally attributed to the accumulation of extracellular amyloid and intraneuronal tau deposition [1]; however, mounting evidence indicates that intracellular accumulation of amyloid-β 42 (Aβ42) is an early pathological marker that precedes neuronal cell death and correlates with cognitive decline. Significant intraneuronal Aβ42 is found in neuronal cell bodies of both AD patients and patients with mild cognitive impairment, a preclinical condition that precedes AD [2], [3]. Animal models for AD corroborate the presence of intraneuronal Aβ42 prior to the appearance of amyloid plaques. Functionally, intraneuronal Aβ42 load is associated with decreased long-term potentiation and increased synaptotoxicity in Aβ42-containing neurons [4], [5], [6]. Since the endosomal/lysosomal pathway plays an important role in mediating intracellular degradation of Aβ, an impairment of this pathway has been suggested as a possible mechanism for increased neuronal Aβ42 load within multivesicular bodies, especially in the postsynaptic compartments [3], [7], [8]. The molecular mechanisms that lead to Aβ42 accumulation during AD are not clear. Recent evidence indicates that autophagy, an upstream branch of the endosomal/lysosomal cascade, is impaired in AD brains and might be responsible for intraneuronal Aβ42 accumulation [9]. In addition to intraneuronal Aβ production, Aβ42 internalized from extracellular space might represent a possible mechanism that contributes to intracellular Aβ42 accumulation [10], [11], [12], [13], [14]. Therefore, understanding the mechanisms of Aβ42 internalization is crucial for defining the neuropathological process of AD and for designing novel methods of AD therapy.

Apolipoprotein E (apoE) secreted by glia in the brain is required for the internalization of lipoproteins by neuronal apoE receptors and plays important roles in brain lipid transport and neuronal repair [15]. It has been suggested that apoE regulates Aβ42 accumulation in neurons during AD. Strong co-immunostaining of intracellular apoE and Aβ42 was evident in APP transgenic mice [16]. The ε4 allele of the *APOE* gene represents the most prevalent genetic risk factor for late-onset AD [17], [18], [19], and apoE4 causes lysosomal leakage when applied exogenously to cultured cells [20], [21]. Furthermore, apoE4 targeted-replacement mice induced to accumulate endogenous Aβ42 show increased neurodegeneration compared to apoE3 mice [22]. Notably, metabolism of both apoE and Aβ42 are regulated by the low-density lipoprotein (LDL) receptor-related protein 1 (LRP1), a large endocytic receptor belonging to the LDL receptor family [15].

LRP1 is synthesized as a 600-kDa precursor protein that interacts with the ER chaperone receptor-associated protein (RAP) [23], [24] and matures in the biosynthetic pathway into an extracellular, ligand-binding subunit of 515-kDa (LRP1-515) and a transmembrane 85-kDa subunit (LRP1-85) that binds several adaptor proteins for efficient endocytic trafficking and signaling [25]. Several lines of evidence suggest a role for LRP1 in AD pathogenesis and in the metabolism of neuronal Aβ42. 1. LRP1 is abundantly expressed in the cell body and in proximal processes of cortical and hippocampal neurons in the brain [26], [27], [28]. 2. LRP1 binds to Aβ42 either directly or via Aβ chaperones such as apoE to mediate brain Aβ clearance [29], [30], [31]. 3. Several genetic risk factors for sporadic AD are ligands of LRP1 which, together with LRP1, are found in senile plaques in AD brains (for reviews see [31], [32]. 4. In AD patients and in elderly people, brain LRP1 levels are significantly decreased and inversely correlate to the age of onset of AD, suggesting that a decrease in LRP1 function might contribute to the cognitive decline [33]. 5. Increased extracellular deposition of Aβ42 was detected in RAP-deficient mice, which display reduced levels of LRP1 [29]. 6. Increased intraneuronal Aβ42 was found in AD model mice overexpressing a functional LRP1 minireceptor, mLRP2 [34]. Together, this evidence indicates a role for LRP1 in the etiology of AD and suggests that LRP1 likely mediates neuronal Aβ42 uptake via receptor-mediated endocytosis. Despite these clues, direct evidence supporting a role for LRP1 in neuronal Aβ42 uptake and accumulation is lacking. Here, using both gain-of-function and loss-of-function approaches, we demonstrate a direct role for LRP1-mediated endocytosis in neuronal Aβ42 uptake and accumulation. These studies define a novel receptor-mediated pathway for Aβ42 entry to neuronal lysosomes, where Aβ42 accumulation may contribute to both Aβ aggregation and neuronal toxicity.

## Results

### Overexpression of LRP1 increases Aβ42 uptake and accumulation in lysosomes in N2a cells

**Figure 1:**
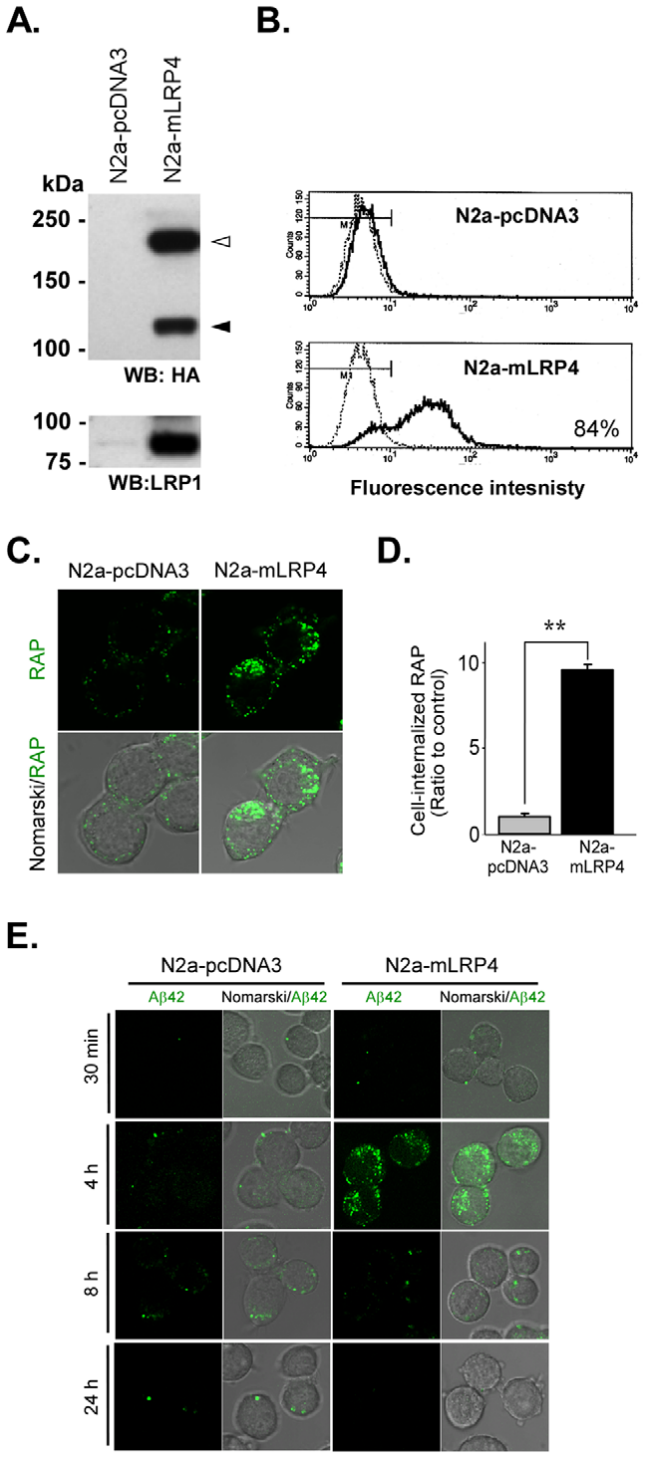
LRP1 minireceptor increases the uptake of Aβ42 in N2a-mLRP4 cells. *A,* cell lysates from N2a cells stably expressing LRP1 minireceptor (N2a-mLRP4) or empty pcDNA3 vector (N2a-pcDNA3) were analyzed by 7.5% SDS-PAGE and Western blotted with anti-HA or anti-LRP1 antibodies. Both the endoplasmic reticulum precursor (*open arrowhead*) and the processed forms after furin cleavage (*closed arrowhead*) are readily detected with anti-HA antibodies. The level of LRP1-85 in N2a-pcDNA3 cells is negligible compared to that in N2a-mLRP4 cells. *B,* flow cytometric analyses of N2a cells stably transfected with pcDNA3 and mLRP4. Negative controls without primary antibody are indicated with *light lines*, whereas the signals from cell surface receptor staining are shown with *dark lines*. 84% of N2a-mLRP4 showed cell surface expression of the LRP1 minireceptor. *C,* LRP1 minireceptor expression increases the endocytosis of RAP in N2a cells. N2a-pcDNA3 and N2a-mLRP4 cells were incubated with 500 nM Alexa488-RAP at 37°C for 30 min, then fixed and analyzed by fluorescence microscopy. *D,* flow cytometric analysis from experiments as in *C.* N2a-pcDNA3 and N2a-mLRP4 cells were detached and additionally treated with pronase to remove cell surface-associated RAP. Quantification of FACS experiments indicates that N2a-mLRP4 cells internalize approximately 8 times more RAP than N2a-pcDNA3 cells. ** p<0.01, *t*-test. n = 3. *E,* LRP1 minireceptor expression increases the endocytosis of Aβ42 in N2a cells. N2a-pcDNA3 and N2a-mLRP4 cells were incubated with 5 µM FAM-Aβ42 for 1 h at 4°C and warmed to 37°C for 0.5, 4, 8 or 24 h, then fixed and examined by confocal microscopy. In N2a-mLRP4 cells, FAM-Aβ42 accumulation peaked 4 h after warming and decreased over time, suggesting that intracellular Aβ42 is eventually delivered for degradation.

To test whether the endocytic activity of LRP1 mediates the uptake of Aβ42 and its accumulation within neurons, we analyzed the uptake of fluorescently labeled Aβ42 in control N2a cells and in N2a cells that overexpress a LRP1 minireceptor, mLRP4. This minireceptor contains the fourth ligand-binding domain of LRP1 and is capable of binding several LRP1 ligands with endocytic properties identical to the full-length LRP1 [35]. We generated stable N2a-mLRP4 and N2a-pcDNA3 cell lines and characterized them for total mLRP4 expression levels by Western blot and FACS (Fig. 1). Our results indicate that functional mLRP4 is properly folded and matured in N2a cells, as evidenced by its furin-induced cleavage into 120- and 85-kDa bands (Fig. 1A). To determine if mLRP4 reached the plasma membrane after synthesis, we analyzed cell surface levels of mLRP4 by FACS in non-permeabilized cells. Approximately 80% of the cells showed expression of cell surface mLRP4 (Fig. 1B). To assess whether cell surface mLRP4 could interact and internalize ligands, we performed uptake experiments with fluorescently labeled RAP. RAP is an LRP1 chaperone of 39-kDa that binds LRP1 with high affinity and is commonly used as a model extracellular ligand for LRP1 [23], [36]. Cell-associated RAP was primarily detected intracellularly, as demonstrated by confocal microscopy (Figure 1C) and increased in N2a-mLRP4 cells compared to N2a-pcDNA3 cells. To quantify the amount of internalized RAP, we performed FACS analysis of similarly treated cultures. Cells were detached and additionally treated with pronase to remove any cell-surface labeled RAP. N2a-mLRP4 cells have an 8-fold increase in the uptake of fluorescently labeled RAP compared to N2a-pcDNA3 cells (Fig. 1D), indicating that mLRP4 expressed in N2a cells is fully functional in mediating ligand internalization. Based on these results, we analyzed the uptake of Aβ42 in N2a-pcDNA3 cells and N2a-mLRP4 cells by performing binding/endocytosis assays and fluorescence microcopy analysis. We choose FAM-Aβ42 as our amyloid-peptide probe since the addition of the carboxyfluorescein moiety at the N-terminal region does not change the properties of Aβ42 aggregation and internalization into neurons [37]. For binding-endocytosis experiments, N2a-pcDNA3 and N2a-mLRP4 cells were first incubated with 5 µM Aβ42 at 4°C for 1 h to allow the binding of Aβ42. Cells were then warmed to 37°C for different periods of time to allow ligand internalization, and analyzed by confocal microscopy as described in the *Experimental Procedures* section. N2a-pcDNA3 cells accumulated a small amount of Aβ42 after 8 h of warming; this accumulated Aβ42 disappeared by 24 h (Fig 1E). In contrast, Aβ42 uptake was faster and accumulation greater in N2a-mLRP4 cells, peaking at 4 h and decreasing over time. These results indicate that increased FAM-Aβ42 uptake occurred in N2a cells overexpressing LRP1 minireceptor and demonstrate that the lysosomal function is unaffected by LRP1 overexpression, given the similar clearance rate of FAM-Aβ42 in the two cell types analyzed.

**Figure 2:**
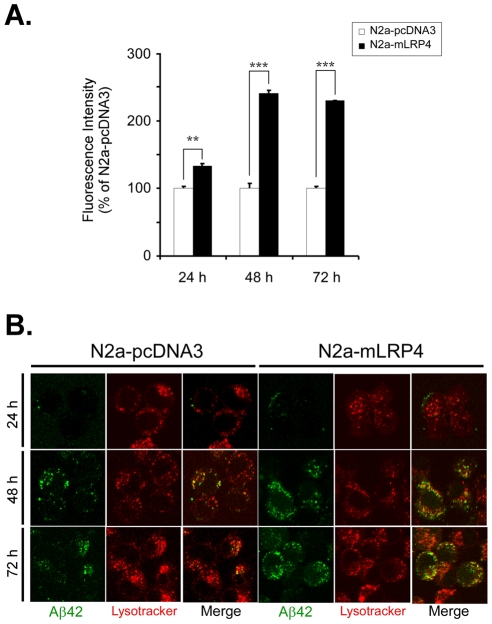
Increased accumulation of intracellular Aβ42 within lysosomes in LRP1 minireceptor-expressing cells. *A,* increased Aβ42 accumulation in LRP1 minireceptor-expressing cells. N2a-mLRP4 and N2a-pcDNA3 cells were treated with 500 nM of FAM-Aβ42 at 37°C for 24, 48 and 72 h, and steady-state levels of intracellular Aβ42 were determined by flow cytometric analyses of pronase-treated cells as described in the *Experimental Procedures*. N2a-mLRP4 cells showed increased Aβ42 accumulation compared to N2a-pcDNA3 cells starting at 48 h of Aβ42 incubation. ** p<0.01, *** p<0.001, *t*-test. n = 3. *B,* increased co-localization of intracellular Aβ42 and lysosomes in N2a-mLRP4 cells. N2a-pcDNA3 and N2a-mLRP4 cells were grown in glass chamber slides and treated with 500 nM of FAM-Aβ42 at 37°C for 24, 48 and 72 h. Lysosomes were labeled with LysoTracker 30 min before the end of each incubation. Cells were then fixed and analyzed by confocal microscopy. Intracellular accumulated Aβ42 was highly co-localized with LysoTracker and increased over time in N2a-mLRP4 cells.

To determine whether internalized Aβ42 is accumulated over time, we performed continuous feeding experiments of FAM-Aβ42 in N2a-pcDNA3 and N2a-mLRP4 cells. The steady-state levels and localization of Aβ42 were determined by flow cytometry and confocal microscopy, respectively (Fig. 2). Cells were incubated with 500 nM FAM-Aβ42 at 37°C for 24, 48 or 72 h and the content of fluorescence per cell was quantified by FACS and presented as a percentage of the levels in N2a-pcDNA3 at each time point (Fig. 2A). Only a slight increase in the levels of FAM-Aβ42 was detected in N2a-mLRP4 cells after 24 h incubation. However, twice the levels were detected at 48 h and 72 h in N2a-mLRP4 cells compared to N2a-pcDNA3 cells, indicating that LRP1 minireceptor overexpression increases the accumulation of FAM-Aβ42 in N2a cells. It has been previously demonstrated that endocytosed Aβ enters the lysosomal pathway in both neuronal and non-neuronal cells, and it has been suggested that both exogenously added Aβ42 and *de novo* synthesized Aβ42 accumulate and aggregate in these organelles [10], [12], [13]. Therefore, we analyzed the subcellular localization of internalized FAM-Aβ42 by confocal microscopy in N2a cells treated as before and analyzed co-localization with lysosomes. For the visualization of acidic organelles, a brief incubation with LysoTracker Red at the end of the experiment was included (Fig. 2B). After 24 h of incubation, a small amount of cell-associated Aβ42 can be detected in N2a-mLRP4 cells. However, this FAM-Aβ42 does not localize into acidic organelles, as evidenced by low co-localization with LysoTracker. However, increased localization of Aβ42 into LysoTracker-positive organelles can be detected in both N2a-pcDNA3 and N2a-mLRP4 cells after 48 and 72 h, and an increased number of double-positive cells were detected in N2a-mLRP4 cells compared to N2a-pcDNA3 cells after 48 h of treatment. Given that pulse-chase experiments showed normal lysosomal activity and increased FAM-Aβ42 internalization in LRP1-expressing cells (Fig. 1E), and because increased steady-state levels of FAM-Aβ42 were found in LRP1-expressing cells at longer incubation times (Fig. 2), these results collectively indicate that LRP1 minireceptor overexpression increases the uptake, delivery and accumulation of Aβ42 into lysosomes in N2a cells.

### LRP1 increases Aβ42 accumulation in GT1-7 and MEF cells

**Figure 3:**
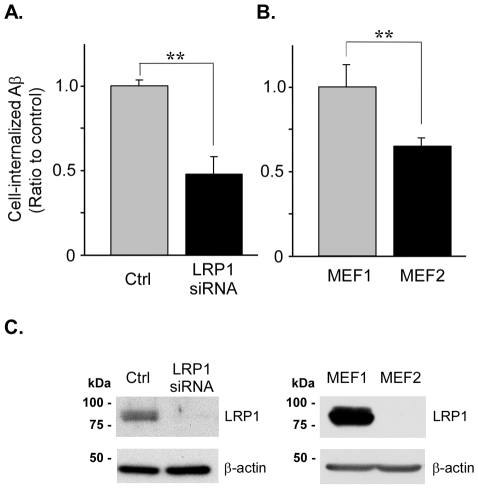
LRP1 mediates internalization and accumulation of Aβ42 in GT1-7 and MEF cells. *A,* LRP1 knockdown decreases Aβ42 accumulation in GT1-7 cells. GT1-7 cells were transiently transfected with LRP1 siRNA or with control, scrambled siRNA. After 72 h, cells were treated with 500 nM FAM-Aβ42 for 4 h and intracellular Aβ42 was determined by flow cytometric analyses of pronase-treated cells as described in the *Experimental Procedures*. *B,* decreased Aβ42 accumulation in mouse embryonic fibroblasts from LRP1 knockout mice. Wild type (MEF1) and LRP1 knockout (MEF2) fibroblasts were treated with 500 nM FAM-Aβ42 for 4 h, and intracellular Aβ42 was determined by flow cytometric analyses. ** p<0.01, *t*-test. n = 3. *C,* cell lysates from GT1-7 and MEF cells treated as in *A* and *B*, analyzed by 7.5% SDS-PAGE, and Western blotted with anti-LRP1 antibodies. Levels of LRP1 were efficiently decreased in both cell types.

To rule out cell type-specific effects and artifacts due to the expression of LRP1 minireceptor, we performed similar FAM-Aβ42 uptake experiments in GT1-7 neuronal cells with reduced endogenous LRP1 levels by siRNA knockdown (Fig. 3A), and in mouse embryonic fibroblasts from both wild-type (MEF1) and LRP1 knockout mice (MEF2) [38] (Fig. 3B). GT1-7 cells were plated and treated as indicated in the *Experimental Procedures* and GT1-7 and MEF cells were incubated with 500 nM of FAM-Aβ42 at 37°C for 4 h. Steady-state levels of intracellular Aβ42 were determined by flow cytometric analysis of pronase-treated cells. A decrease in the internalization of FAM-Aβ42 was observed in GT1-7 cells with decreased LRP1 levels, and in MEF2 cells compared to MEF1. Depletion of LRP1 levels in both knockdown and knockout cells were confirmed by Western blot (Fig. 3C). Collectively, these results indicate that LRP1 expression directly impacts the internalization of FAM-Aβ42 in both neuronal and non-neuronal cells.

### LRP1 endocytosis is required for Aβ42 accumulation in neuronal cells

**Figure 4:**
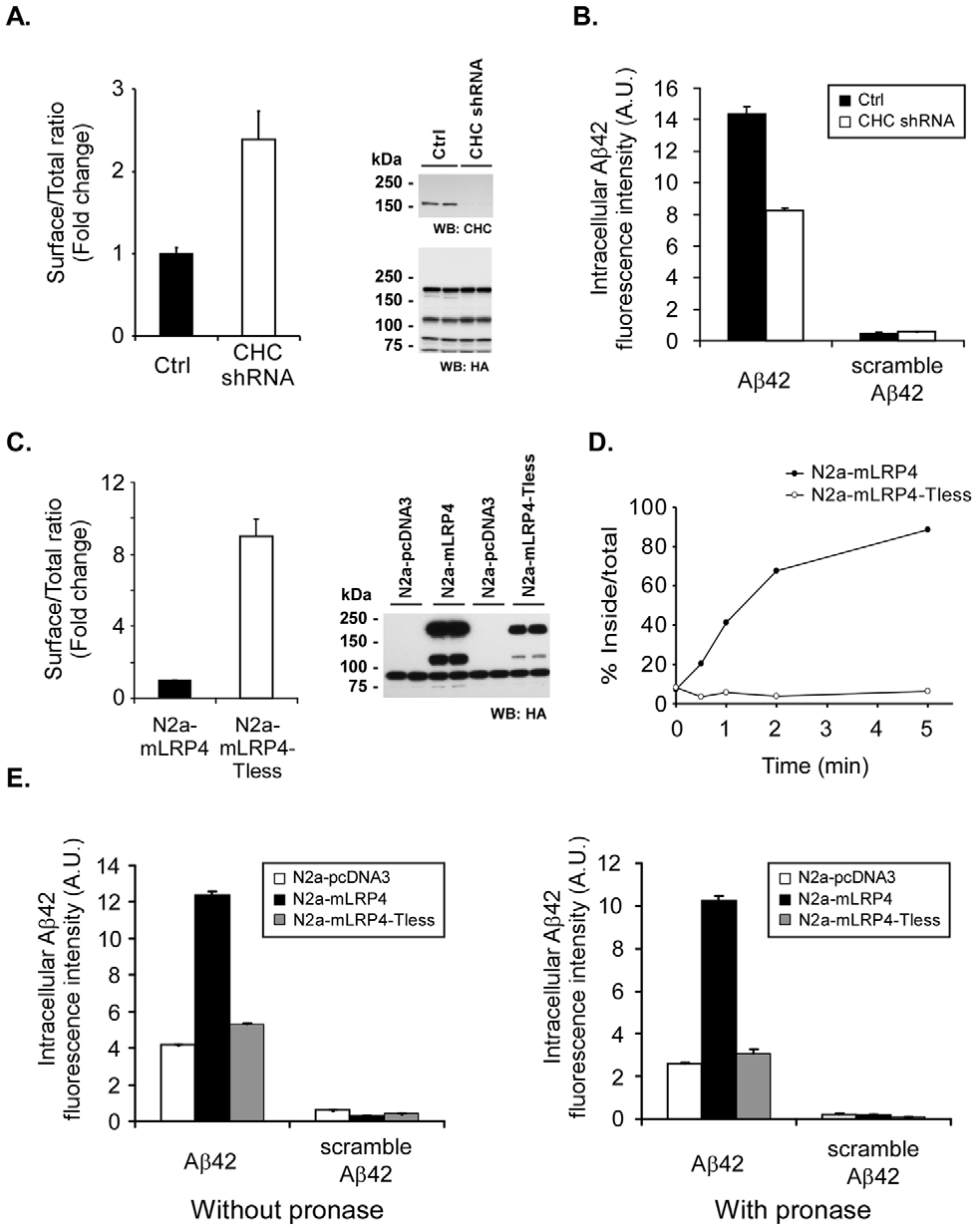
LRP1 endocytosis is required for Aβ42 uptake and accumulation in N2a cells. *A,* clathrin heavy chain (CHC) knockdown increases cell surface levels of mLRP4. N2a-mLRP4 cells were infected with CHC shRNA lentivirus or pLKO, control lentivirus. The levels of cell surface and total pools of mLRP4 were determined by flow cytometric analyses with anti-HA antibody in non-permeabilized and saponin-treated cells, respectively. The surface-to-total ratio were calculated and plotted as fold-change to control-infected cells. *Right panel*, decreased CHC levels and normal mLRP4 levels in transduced N2a-mLRP4 cells were verified by Western blot from sister cultures. *B,* CHC knockdown decreases accumulation of Aβ42 in N2a-mLRP4 cells. N2a-mLRP4 cells infected with clathrin heavy chain lentivirus as in *A*) were treated with 500 nM of FAM-Aβ42 or the corresponding control, scrambled peptide for 48 h. The intracellular level of FAM-Aβ42 was determined by flow cytometric analyses of pronase-treated cells. *C,* deletion of LRP1 tail increases cell surface levels of mLRP4. The cell surface and total pools of the LRP1 minireceptor were determined by flow cytometric analyses with anti-HA antibody as in *A* in N2a-mLRP4 cells and in N2a cells stably transfected with a deletion variant lacking the cytoplasmic tail of mLRP4 (mLRP4-Tless). The surface-to-total ratios were then calculated and plotted as fold-change to N2a-mLRP4 cells. *Right panel*, HA blot showing the expression level of minireceptors in N2a stable cell lines. *D,* deletion of LRP1 tail decreased the mLRP4 endocytosis rate in N2a cells. N2a-mLRP4 and N2a-mLRP4-Tless cells were incubated with 5 nM ^125^I-RAP at 4°C for 60 min, and then shifted to 37°C for the indicated times. At each time point, the amounts of ligand that is either internalized or that remains at the cell surface were determined and the ratios of internalized to total cell-associated ligand were plotted against time. Values are the average of triple determinations with the S.D. indicated by *error bars*. *E*, impaired LRP1 endocytosis rate decreases accumulation of Aβ42 in N2a-mLRP4 cells. N2a-pcDNA3, N2a-mLRP4 and N2a-mLRP4-Tless cells were treated with 500 nM of FAM-Aβ42 or the corresponding control, scrambled peptide for 48 h and the cell-associated (without pronase) and intracellular (with pronase) levels of Aβ42 were determined by flow cytometric analyses.

To determine whether the endocytic function of LRP1 is required for Aβ42 internalization and accumulation, we first analyzed the effect of clathrin knockdown on the uptake of FAM-Aβ42 in N2a-mLRP4 cells. We decreased the expression of clathrin heavy chain by lentiviral-mediated delivery of small hairpin interfering RNA (shRNA) in N2a-mLRP4 cells and analyzed cell surface levels of mLRP4 by FACS. Lentiviral-mediated knockdown of clathrin heavy chain substantially decreases the protein levels of clathrin heavy chain and evokes a twofold-increase in the levels of cell surface mLRP4 in N2a cells (Fig. 4A), consistent with an impairment of clathrin-mediated endocytosis of this LRP1 minireceptor. We therefore tested the uptake of FAM-Aβ42 in similarly infected cultures. Approximately a twofold decrease in FAM-Aβ42 uptake was observed in clathrin knocked down cells compared to cells infected with a control, empty lentivirus (Fig.4B). As a control for specificity, we analyzed the uptake of a FAM-Aβ42 peptide that contained the same amino acid composition but in a scrambled sequence. Very little uptake of fluorescently labeled scrambled FAM-Aβ42 was detected in both control and clathrin knockdown N2a-mLRP4 cells, indicating that Aβ42 internalization requires the intact sequence of Aβ42. Together, these results indicate that clathrin-mediated endocytosis is a critical pathway for Aβ42 uptake and strongly suggest that neuronal Aβ42 uptake is a receptor- mediated process that requires the endocytic properties of LRP1. To further confirm this, we specifically targeted the endocytic function of mLRP4 and analyzed the internalization of FAM-Aβ42 in LRP1 endocytosis-deficient cells. The molecular determinants of LRP1 endocytosis reside in its cytoplasmic tail [39], [40]. We have previously demonstrated that, despite reaching the plasma membrane, a deletion of the complete cytoplasmic tail of mLRP4 (mLRP4-Tless) impairs its endocytic function [34]. We therefore prepared N2a-mLRP4-Tless stable cell lines and first characterized the endocytic properties of the LRP1 minireceptor in this neuronal cell type. Flow cytometric analysis indicates that deletion of the mLRP4 cytoplasmic tail increases its distribution at the cell surface (Fig. 4C), consistent with impaired endocytosis. Additionally, the endocytosis rate for ^125^I-RAP was decreased in these cells (Fig. 4D). We compared the uptake of FAM-Aβ42 in N2a-pcDNA3, N2a-mLRP4 and N2a-mLRP4-Tless by FACS in both pronase-treated and untreated cells. Again, cell-associated FAM-Aβ42 increases in N2a-mLRP4 cells compared to N2a-pcDNA3 cells. Notably, FAM-Aβ42 uptake was similar in N2a-mLRP4-Tless and N2a-pcDNA3 cells, consistent with a role for LRP1 endocytosis in Aβ42 uptake (Fig. 4E). Therefore, impairing the endocytic function of mLRP4 by clathrin knockdown or by the removal of its cytoplasmic tail decreased FAM-Aβ42 uptake, demonstrating that LRP1 endocytosis is required for Aβ42 internalization in N2a cells.

### Increased cellular toxicity in N2a-mLRP4 cells incubated with Aβ42

**Figure 5:**
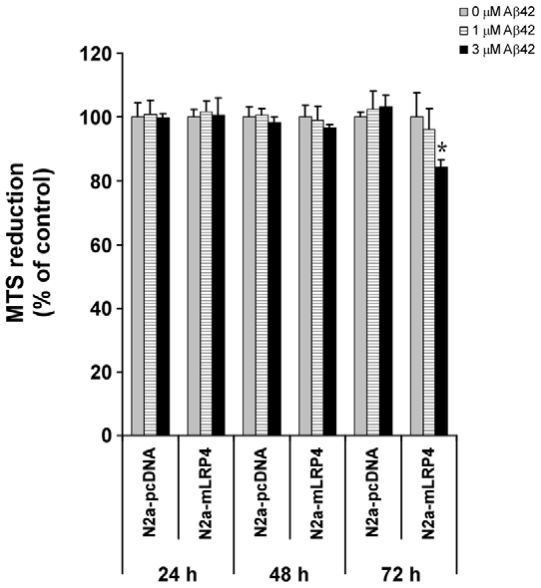
Increased susceptibility to Aβ42-mediated cell death in LRP1 minireceptor-expressing cells. N2a-pcDNA3 and N2a-mLRP4 cells were incubated with increasing concentrations of Aβ42 for 24, 48 and 72 h and cell viability was assessed by the reduction of the MTS dye. Decreased viability was detected only after 72 h incubation with a high concentration of Aβ42 in LRP1 minireceptor expressing cells. * p<0.05, *t*-test. n = 3.

It has been previously demonstrated that increased delivery of Aβ42 into lysosomes increases leakage of the lysosomal contents into the cytosol and hence induces cell toxicity [20], [21], an effect that is increased by apoE4 and might depend on LRP1 function. We hypothesized that Aβ42 treatment might increase cell death in mLRP4-overexpressing N2a cells because we detected increased co-localization of Aβ42 with lysosomes in these cells. We treated N2a-pcDNA3 and N2a-mLRP4 cells with Aβ42 for 24, 48 and 72 h and assessed cell viability by reduction of the MTS redox dye. Aβ42 was prepared identically to the FAM-Aβ42 experiments. A slight yet significant decrease in cell viability was detected only in mLRP4- expressing cells after 72 h of incubation with 3 µM Aβ42, suggesting that LRP1-mediated delivery of Aβ42 into lysosomes might not be sufficient to trigger cell death in N2a cells at early time points or at lower Aβ42 concentrations (Figure 5). Together, our results demonstrate that LRP1 mediates neuronal endocytosis of Aβ42, which leads to eventual lysosomal accumulation at high Aβ42 concentrations and slightly decreases neuronal viability.

## Discussion

The importance of LRP1 in Aβ metabolism has been recognized because of its ability to mediate brain efflux at the blood-brain barrier and because of its role in modulating APP processing to Aβ[31]. Here, we show that LRP1 endocytosis plays an additional role in Aβ42 uptake and delivery into intracellular compartments of neuronal cells. A faster transient accumulation of intracellular Aβ42 was detected in N2a-mLRP4 cells compared to N2a-pcDNA3 cells after 4 h of endocytosis in binding-endocytosis experiments (Fig. 1E). However, continuous uptake experiments suggest that, over time, Aβ42 might escape the degradation pathway and start to accumulate, which is evident after 48 h of incubation (Fig. 2B). It is interesting that N2a-pcDNA3 cells showed a delayed accumulation of Aβ42 in binding-endocytosis experiments assays, with a peak at 8 h after warming. Because N2a cells express very low levels of endogenous LRP1 (Fig. 1A, anti-LRP1 blot), and given that endogenous LRP1 and the mLRP4 minireceptor possess similar kinetics of endocytosis, these results suggest that an LRP1- independent pathway is responsible for Aβ42 internalization in N2a-pcDNA3 cells, which mediate slower kinetic delivery into lysosomes. The LRP1 minireceptor mLRP4 might compete for these Aβ42 cell surface binding sites and give rise to the rapid internalization observed in N2a-mLRP4 cells. Notably, at 8 h after warming up, N2a-mLRP4 cells exhibited decreased levels of Aβ42 in our imaging experiments, indicating that the degradation of Aβ42 by the LRP1-dependent pathway is very efficient. The mechanisms that are responsible for Aβ42 accumulation in N2a-mLRP4 cells in the continuous endocytosis experiments are unknown. However, it is tempting to speculate that the constant presence of relatively high concentration of Aβ42 leads to high Aβ42 concentrations in the lysosomes that might exceed its degradation capacity. Our data demonstrate that the endocytic activity of LRP1 is required for Aβ42 internalization and accumulation because both clathrin heavy chain knockdown and deletion of the LRP1 tail decreased the levels of intracellular Aβ42.

Previous reports addressed the question of whether LRP1 mediates the uptake of Aβ into neurons. Experiments performed in cortical neurons from Tg2576 APP transgenic mice suggested that LRP1 might endocytose and degrade endogenously produced Aβ complexed with LRP1 ligands α2M and lactoferrin [41]. However, these experiments relied primarily on the use of RAP to define the LRP1 pathway. Because RAP also inhibits binding of ligands to several other members of the LDLR family, the specific function of LRP1 in these studies was not clear. Endogenously produced Aβ adds a second level of complexity to that study [41] as Aβ might be either produced in the endocytic compartments or trafficked there directly without secretion. Similarly, the uptake of Aβ40 complexed with apoE has been previously characterized by FACS into purified rat brain synaptosomes, but again only RAP was used to define the LRP1 pathway [42]. To our knowledge, our study is the first to report changes in Aβ trafficking by direct targeting the expression and endocyitic function of LRP1 in neural cells. We show that in neural cells, overexpressing LRP1 minireceptor increases Aβ42 internalization and accumulation, whereas knockdown of endogenous LRP1 decreases these processes. These findings establish a direct role for LRP1 in neuronal Aβ42 uptake, degradation and/or accumulation. It is worth mentioning that induction of Aβ accumulation in the brains of apoE-targeted replacement mice through inhibition of the extracellular Aβ protease neprilysin [22] showed that Aβ accumulates intracellularly along with apoE in CA1 and septal neurons of apoE4 targeted-replacement mice, and that this effect correlates with increased LRP1 levels in these neurons [22], [43]. This *in vivo* evidence is in line with our current findings in cultured neurons and our previous studies in mouse brain [34] which demonstrated that LRP1 function is critical for the uptake and accumulation of Aβ42. Since apoE3 and apoE4 bind similarly to LRP1 [44], additional factors might account for the apoE isoform-specific effects on neuronal Aβ42 accumulation. We are currently evaluating the contribution of differential trafficking of apoE isoforms in neuronal Aβ42 metabolism and accumulation.

Kim *et al.* have recently demonstrated that LDLR, the founding member of the LDLR gene family to which LRP1 belongs, negatively regulates apoE levels in the brain and promotes Aβ clearance *in vivo* [45]. They elegantly showed that as little as a twofold increase in brain LDLR decreases apoE levels and enhances the elimination of extracellular Aβ from the brain interstitial fluid, with a concomitant decrease in amyloid plaque formation. Although it remains to be established whether the effect of LDLR on Aβ metabolism is apoE dependent, this finding identifies another apoE endocytic receptor that modulates Aβ metabolism in the brain. It is interesting that LDLR and LRP1 have overlapping binding properties for apoE3 and apoE4; however, LRP1's high expression level in the brain and fast endocytosis rate likely make this receptor an important candidate for Aβ metabolism in the brain. Supporting this hypothesis, we have previously shown that LRP1 knockout in forebrain neurons leads to increased brain apoE levels in mice [46]. On the other hand, Aβ42 immunoreactivity was significantly decreased in PDAPP mice lacking apoE, supporting a role for apoE in mediating Aβ uptake *in vivo* [34]. More detailed characterization of APP, apoE, LDLR and LRP1 is required to define the precise interplay among these lipid metabolic proteins.

The increased accumulation of Aβ42 in lysosomes of LRP1-expressing cells (Fig. 2B) and its correlation with decreased cell viability (Fig.5) is intriguing. Imaging experiments of endocytosed Aβ42 suggest that a large portion of LRP1-delivered Aβ42 is degraded. However, when the amount of Aβ42 exceeds lysosomal degradation capacity, Aβ42 likely accumulates and aggregates. It has been demonstrated that localization of Aβ42 into lysosomes is toxic to neurons, in particular in the presence of apoE4 [20], [21], [22]. The acidic environment of the lysosomal compartment might be key for the potentiating effect of apoE4 on Aβ42-induced lysosomal leakage and apoptosis, as demonstrated by Ji *et al.* Notably, this group found that reducing LRP1 levels by siRNA knockdown decreases the stimulatory effects of apoE4 [21], which is consistent with our finding that LRP1 mediates endocytosis and lysosomal trafficking of Aβ42 in neuronal cells. In addition, it has been demonstrated that LRP1 plays a role in the TGFβ-induced, Aβ42-mediated lysosomal leakage [47], [48]. Our neural viability experiments showed that cell viability was slightly decreased after 72 h of incubation with a high concentration of Aβ42 in LRP1-expressing cells (Fig. 5). It will be of great interest to evaluate whether increased co-localization of Aβ42 with lysosomes in apoE4-treated N2a-mLRP4 cells leads to more severe lysosome-associated neuronal toxicity in our cell-based system.

Increased accumulation of intracellular Aβ has various pathological effects on cell and organelle function, including proteasome inhibition, mitochondrial abnormalities, tau hyperphosphorylation and presumably, the seeding of amyloid plaques after neuronal cell death [49]. Immunohistochemical analyses of AD brains strongly suggests that amyloid plaques arise when an Aβ-accumulating neuron undergoes a single lysis event [50], [51]. In support of this, 3xTg-AD mice treated with anti-Aβ antibodies show a parallel decrease of extracellular amyloid deposits and intraneuronal Aβ in pyramidal cells, which suggests there is a dynamic relationship between the intracellular and extracellular pools of Aβ [52]. Therefore, it is conceivable that the endocytic activity of LRP1 might contribute to the appearance of plaques by increasing the internalization of Aβ, with a consequent decrease in neuronal function and viability and release of amyloid seeds into the brain parenchyma. Our LRP1-overexpressing mouse model, however, did not show an increase in the amyloid plaque burden when crossed to PDAPP amyloid model mice [53]. Additional compensatory mechanisms mediated by LRP1, including the activation of survival signaling pathways, might prevent the adverse effects of intraneuronal Aβ accumulation *in vivo* and its subsequent release. It is worth mentioning that LRP1 is indeed a signaling receptor capable of activating survival pathways including ERK, Akt and β-catenin [54], [55], [56], [57], [58]. Among these pathways, Akt is regulated by LRP1 in differentiated neurons and targeted by intraneuronal Aβ[57], [59]. We propose that dysregulated neuronal functions of LRP1 in Aβ production, clearance, as well as cell survival might contribute to the neuronal pathology in AD.

In conclusion, we show that modulation of LRP1 expression or its endocytic function directly impacts neuronal Aβ42 uptake, degradation and/or accumulation. These results provide strong evidence that an LRP1-mediated pathway is crucial for Aβ42 trafficking and metabolism in neurons.

## Materials and Methods

### Reagents and Antibodies

FAM-Aβ42 peptide (23526) and scrambled FAM-Aβ42 peptide (60892) were from Anaspec (Fermont, CA). Unlabeled Aβ42 was obtained from Bachem (Torrance, CA). Anti-HA monoclonal antibody (12CA5) was from Babco (Richmond, CA). Anti-Clathrin Heavy Chain monoclonal antibody was from Sigma (St Louis, MO). Anti-LRP1-85 was a rabbit polyclonal antibody produced in-house. High glucose Dulbecco's modified Eagle's medium (DMEM, D-5796), fetal bovine serum, G418, sodium azide, CaCl_2_, cell dissociation solution, pronase, PMSF, saponin and 1,1,1,3,3,3-hexafluoro-2-propanol (HFIP; catalog number H8508) were from Sigma (St Louis, MO). OptiMEM-I, Lipofectamine 2000 and LysoTracker® Red DND-99 were from Invitrogen (Carlsbad, CA). Immobilon-P PVDF membrane was from Millipore (Bedford, MA). 8-well Lab-Tek chambers (catalog number 155411) were from Nunc (Rochester, NY). Complete EDTA free cocktail of protease inhibitors was from Roche Applied Sciences (Indianapolis, IN).

### Cell culture and stable cell lines

Neuro-2A (N2a) is a mouse neuroblastoma cell line available from the American Type Culture Collection (CCL-131). Subconfluent N2a cells were maintained in growth media (1:1 DMEM:OptiMEM-I mixture containing 5% FBS and 2 mM L-glutamine). For generation of stable cell lines, N2a cells were transfected with Lipofectamine 2000 and selected in growth media supplemented with 800 µg/mL G418 for at least 2 weeks until the verification of transgene expression. The immortalized hypothalamic neuronal cell line GT1–7 [60] and MEF cells were grown in DMEM media containing 10% FBS and 2 mM L-glutamine. For all the experiments, cells were kept at low passage number (<10) and were routinely analyzed for *Mycoplasma* contamination using the MycoSensor PCR assay kit (Stratagene, LaJoya, CA).

### FACS analysis of surface and total ratio and purity of clones

Sub-confluent N2a cells in 100 mm Petri dishes were washed twice in PBS and non-enzymatically detached by incubating 5 min at 37°C in cell dissociation solution. After washing in growth media, samples were divided in two parts and half of the samples were kept in a minimal volume of PFN buffer (PBS supplemented with 1.5% FBS and 0.1% sodium azide) for the non-permeabilized group. The remaining cells were resuspended in PFN containing 0.05% saponin and gently rocked for 30 min at 4°C. After washing with growth media, permeabilized and non-permeabilized cells were incubated with 50 µg/mL anti-HA antibody for 2 h at 4°C. After two washes with PFN, cells were incubated with 50 µg/mL goat anti-mouse-Ig fluorescein isothiocyanate as a secondary antibody and similarly incubated for 45 min. Cells were washed twice, fixed in PFN-1% PFA and analyzed in a FACSCalibur cytometer (BD Bioscience) equipped with an argon ion laser. Laser excitation of 488 nm for fluorescein isothiocyanate was used. Twenty thousand cells from each sample were analyzed and histograms were generated using CellQuest software. After the subtraction of blank controls lacking primary antibody, the geometric means of fluorescence intensity were obtained for non-permeabilized and permeabilized samples, representing surface and total minireceptor levels, respectively. The surface-to-total ratio was calculated and utilized for comparison purposes. Unpermeabilized conditions were used to determine the purity of stable cell lines.

### Immunoblot Analysis

After indicated treatments, cells were washed twice in PBS and cell lysates were prepared in PBS containing 1% TX-100, supplemented with 1 mM PMSF and a protease inhibitor cocktail. After 30 min on ice, cells were briefly vortexed and cell debris discarded by centrifugation at 10,000× g for 10 min at 4°C. 20 µg of protein was resolved by reducing 10% SDS-PAGE and transferred to PVDF membranes overnight. After the membranes were blocked, proteins were detected by using 1∶1,000 dilution of primary antibodies for commercial antibodies or 1 µg/mL of purified anti-LRP polyclonal IgG. The immunoblotting was followed by detection with a horseradish peroxidase-conjugated secondary antibody and enhanced chemiluminiscence substrate (GE Healthcare, Uppsala, Sweden). Bands on films were scanned using Quantity-One software (Bio-Rad, Hercules, CA).

### Preparation of Aβ42 and RAP

Synthetic FAM-Aβ42 was resuspended essentially as described by Stine *et al.* to yield an essentially assembly-free/monomeric preparation [61]. Briefly, 0.1 mg vial of FAM-Aβ42 was resuspended in HFIP, divided into aliquots and dried in a current of nitrogen gas. FAM-Aβ42 and Aβ42 protein films were thoroughly resuspended in DMSO at 5 mM and immediately diluted with water to form a 100 µM Aβ42 stock solution. To avoid aggregation of the peptides during treatment of cells, resuspended aliquots were used within 30 min after resuspension. RAP protein was routinely produced in our lab and was labeled with an Alexa-488 labeling kit (Millipore, Bedford, MA) and stored at −80°C until use [23].

### *In vitro* FAM-Aβ42 uptake and accumulation assays by laser scanning confocal microscopy

For the imaging experiments, stable N2a cells were plated into an 8-well chambered coverglass at 16,250 cells/cm^2^ with one independent chambered coverglass for each time point, and cells were grown overnight. For uptake experiments, cells were washed three times with cold DMEM and binding of 5 µM FAM-Aβ42 was performed in DMEM at 4°C for 1 h. After binding, unbound FAM-Aβ42 was removed by three washes with cold PBS. To initiate the uptake, cells were switched to 37°C for different time periods by adding pre-warmed internalization media (DMEM containing 10% FBS and 2 mM L-glutamine) and bringing the chambered coverglass back to the incubator until completion of the kinetic time point. For steady-state accumulation experiments, cells were washed twice with PBS and incubated for the indicated periods of time with pre-warmed internalization media containing 500 nM FAM-Aβ42. In some experiments, 100 nM LysoTracker Red probe was added 30 min prior to the end of the experiment. Cells were then fixed and analyzed by a confocal laser scanning microscope (Fluoview 500, Olympus) using a 40x objective lens. The signal corresponding to Aβ42 was visualized in the fluorescein channel and LysoTracker was visualized in the Alexa-569 channel.

### Quantification of Aβ42 accumulation by FACS analysis

For quantification of Aβ uptake experiments, stable N2a cells were plated into 12-well plates at 16,250 cells/cm^2^ and grown overnight. Similar to imaging assays, cells were washed twice with PBS and incubated for the indicated periods of time with pre-warmed internalization media containing 500 nM FAM -Aβ42. After incubation, cells were washed twice with PBS and non-enzymatically detached as before in 500 µL cell dissociation solution. Cells were sequentially washed once with 1 mL of cold internalization media and twice with 2 mL cold PFN. Cells were finally resuspended in PFN-1% PFA and analyzed by FACS as before. For MEF-1 and MEF-2 experiments, 5,000 cells/cm^2^ were plated in 12-well plates the day before. Cells were then washed twice with PBS and incubated 4 h with pre-warmed internalization media containing 500 nM FAM-Aβ42. After completion of the incubation time, cells were processed by FACS analysis as described above for N2a cells.

### Kinetic Analysis of Endocytosis

Kinetic analysis of receptor-mediated endocytosis was carried out as described previously [40]. Briefly, stably transfected N2a cells were plated at a density of 2×10^5^ cells/well in a 12-well plate and used after overnight culture. Cells were rinsed twice with cold PBS and then incubated in 0.5 ml ice-cold ligand binding buffer (DMEM-5 mM CaCl_2_ containing 0.6% bovine serum albumin) with 5 nM ^125^I-RAP. The binding of iodinated proteins was carried out at 4°C for 60 min with gentle rocking. Unbound ligand was removed by washing cell monolayers three times with cold binding buffer. Ice-cold stop/strip solution (0.2 M acetic acid, and 0.1 M NaCl, pH 2.6) was added to one set of plates without warming and kept on ice. The remaining plates were then placed in a 37°C water bath and 0.5 ml of pre-warmed ligand binding buffer was quickly added to the cell monolayers to initiate internalization. After each time point, the plates were placed on ice, and the ligand binding buffer was replaced with ice-cold strip/stop solution. Ligand remaining on the cell surface was stripped by incubation of cell monolayers with ice-cold stop/strip solution for 10 min and counted. The sum of internalized and cell-surface ligand after each assay was used as the maximum potential internalization. The fraction of internalized ligand at each time point was calculated and plotted.

### LRP1 and clathrin heavy chain knockdown

LRP1 knockdown was carried out in GT1-7 cells by siRNA transfection using Lipofectamine 2000 (Invitrogen, Carlslab, CA) and 120 nM of dsRNA duplex (Ambion, Austin, TX). Briefly, cells were plated in 6-well plates the day before transfection and grown overnight. Scrambled and LRP1 siRNA duplexes were generated according to the manufacturer's instruction and complexed with Lipofectamine 2000 as previously described [62]. FAM-Aβ42 experiments were performed 72 h after siRNA transfection and analyzed as previously described for N2a cells. For clathrin heavy chain knockdown, shRNA lentiviruses were produced in the Viral Vectors Core facility at Washington University School of Medicine. In brief, 293T cells were transfected with pLKO.1-derived constructs together with the pHR'8.2ΔR and pCMV-VSV-G packaging systems as previously described [63]. Conditioned media were concentrated by ultracentrifugation, titrated against HT1080 cells and kept frozen in 20 µL aliquots at −80°C until use. N2a cells were plated at 16,250 cells/cm^2^ in 100 mm Petri dishes and grown overnight. N2a cells were infected two times by incubating the cultures with 1×10^7^ transforming units of lentivirus overnight. After the second infection, the cells were split into 12-well plates for Western blot and FAM-Aβ42 accumulation experiments or were kept an additional day without being split up for cell surface and total mLRP4 levels determination by FACS.

### MTS redox assay in Aβ42 treated cells

N2a-pcDNA3 and N2a-mLRP4 cells were plated at 16,250 cells/cm^2^ in 48-well plates and grown overnight. Serial dilutions of freshly prepared synthetic Aβ42 were prepared by diluting the Aβ42 stock with internalization media. Culture media was then replaced with media containing 0, 1 or 3 µM Aβ42 and cells were incubated for an additional 24, 48 or 72 h. Cell viability was measured by using one-solution cell titer kit at each corresponding time point (Promega, Madison, WI). After 4 h labeling, the soluble reduced formazan product was quantified by directly reading the plate in a Bio-Tek plate reader. After background subtraction from media-only containing wells, results were represented as % of the corresponding control and untreated N2a cells.
